# Supplementary material for: ANOVA Design for the Optimization of TiO2 Coating on Polyether Sulfone Membranes
Source: Molecules. 2019 Aug 12;24(16):2924. doi: 10.3390/molecules24162924 (PMC6720000; doi:10.3390/molecules24162924)
Supplement: Supplementary file 1 [file molecules-24-02924-s001.pdf]

Appendix:

Table A.1. Materials for making the polymeric membrane

Material	Molecular Weight	Company	Batch Number
NMP (1-Methyl-2-Pyrolidone) as Solvent	99.13	Scharlav Chemie S.A.	MED4942500
DMAc as Solvent	87.12	Scharlav Chemie S.A.	-
Isopropanol as non-solvent	60.10	Ajax Finechem Pty Ltd	0809287
Glycerol for post-treatment	92.09	Ajax Finechem Pty Ltd	0810081
PVP40T (Polyvinylpyrrolidone) as pore former	40000	Sigma Aldrich	098K0011
Polyethersulfone (PES) as polymer	58000 gr/mol	BASF Co.Ltd	Unavailable
Pluronic F 127	2200 Da	Sigma Aldrich	9003-11-6
IM 22	82.10	Sigma Aldrich	M50850
PEG	100	Sigma Aldrich	25322-68-3
TTIP 97%	284.22 g/mol	Sigma Aldrich	546-68-9
BSA(bovine serum albumin)	67	Morrgate Biotech	9048-46-8
Ethanol (anhydrous)	-	Ajax Finechem Pty Ltd	A4503
Perchloric acid	-	G. Frederick Smith Chemical Co.	7601-90-3
2,4-Pentanedione	114.14	Lancaster	815-57-6

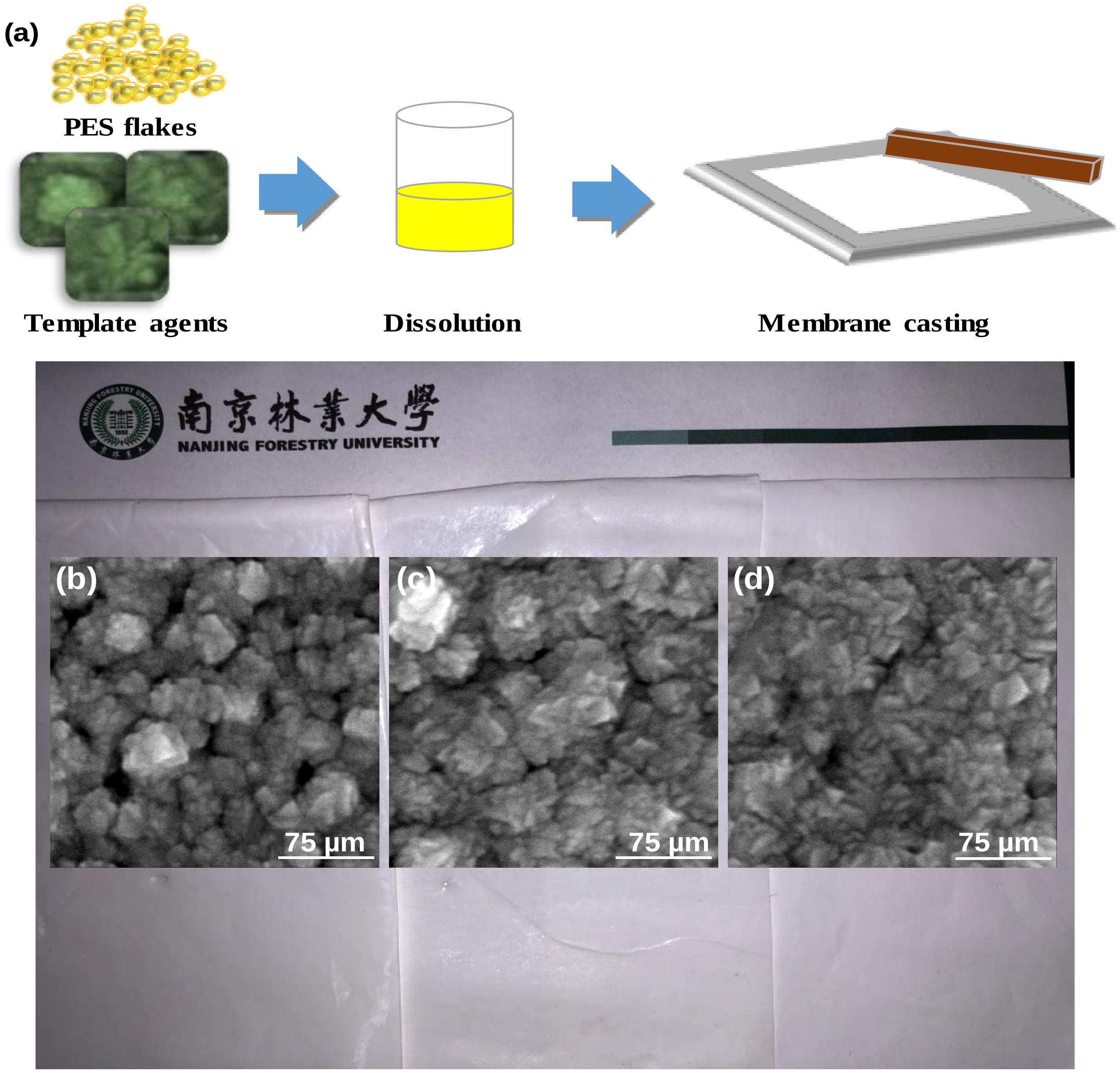

Figure A.1. a) Scheme for preparation of membrane b-d) digital image of membrane and morgholigical effect of IM22, PEG and F127, repectively.

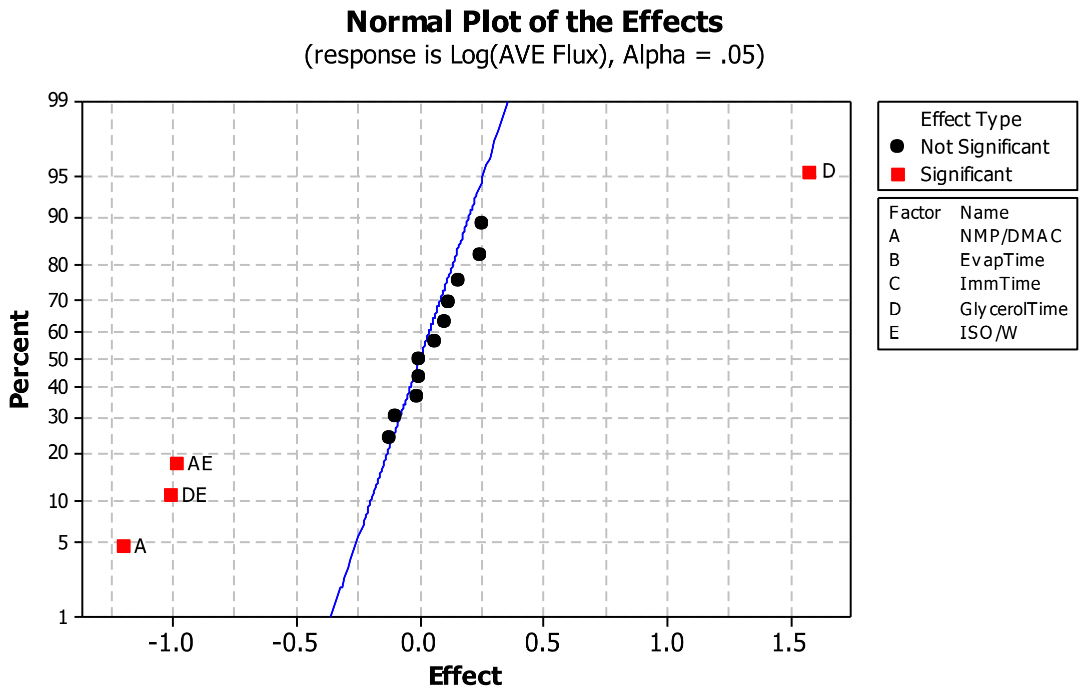

Figure A.2 Normal probability plot of estimated effects and interactions on flux

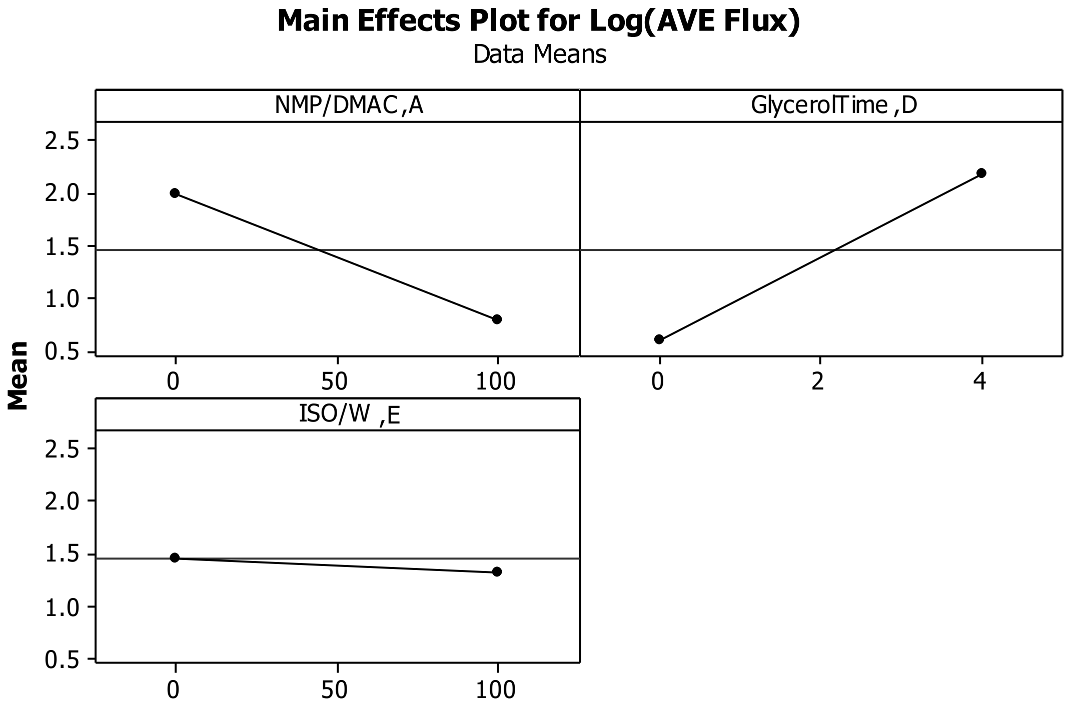

Figure A.3 Main effects plots of response (Log (Flux))

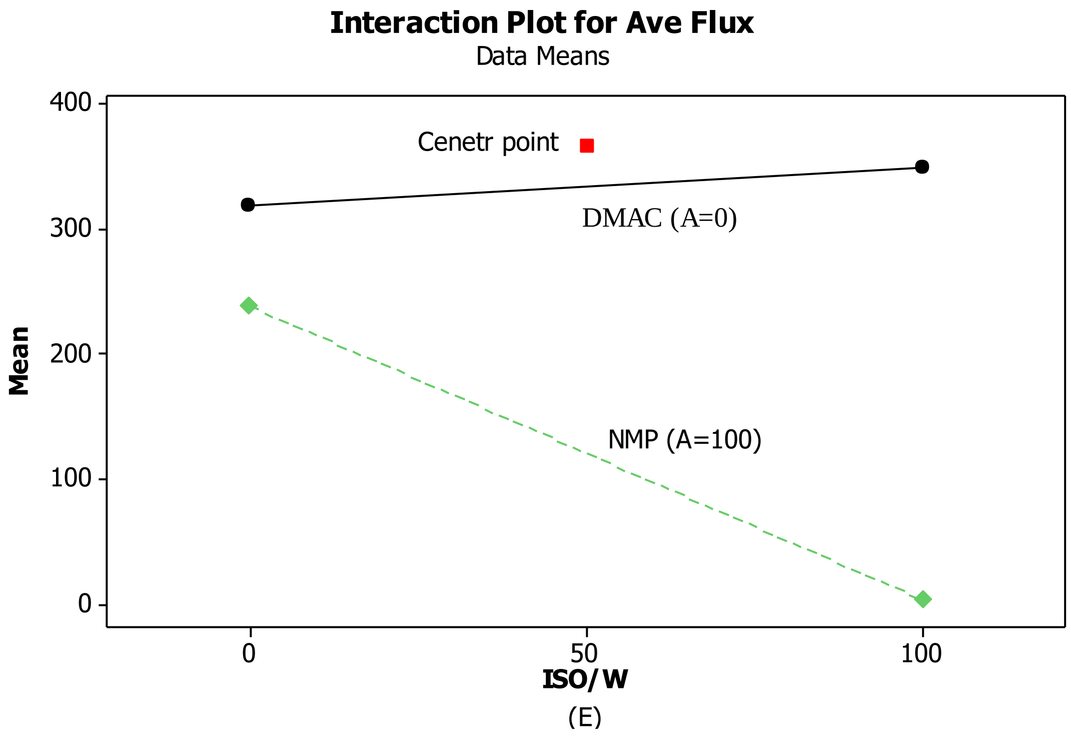

Figure A.4 The interaction of A and E on average Flux

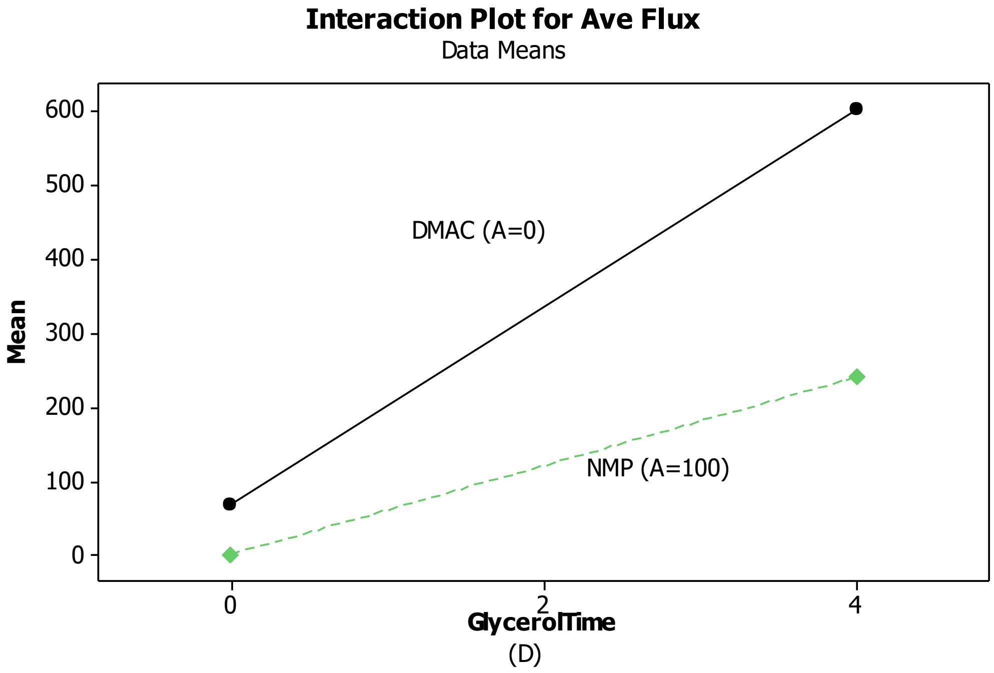

Figure A.5 The interaction of A and D on average Flux

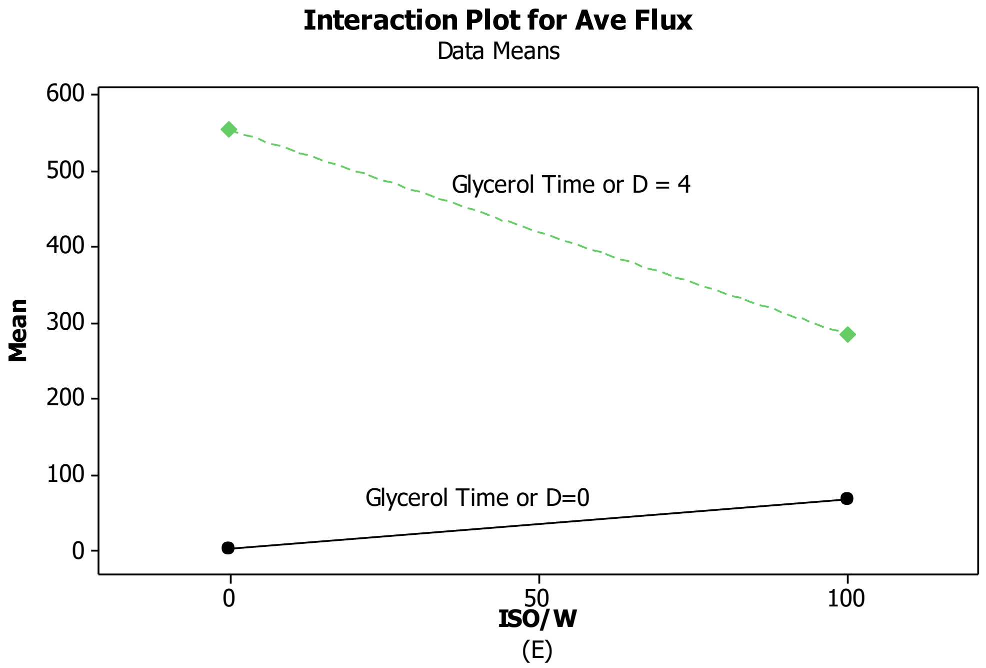

Figure A.6 The interaction of D and E on average Flux

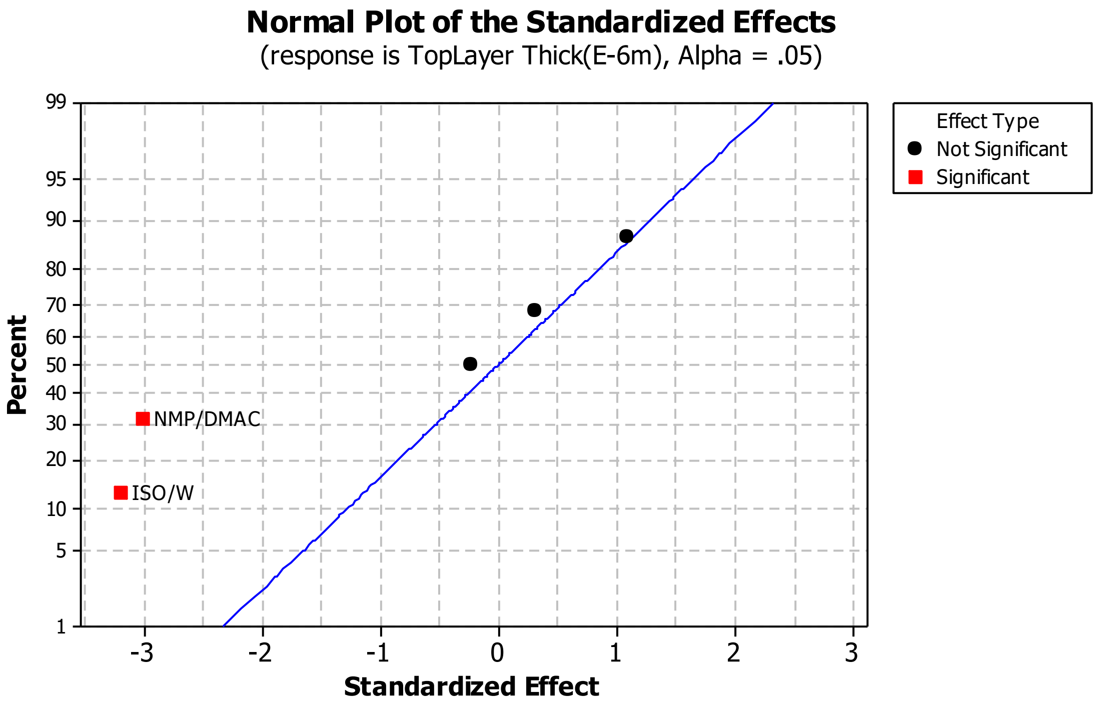

Figure A.7 Normal probability plot of estimated main effects on Top layer thickness

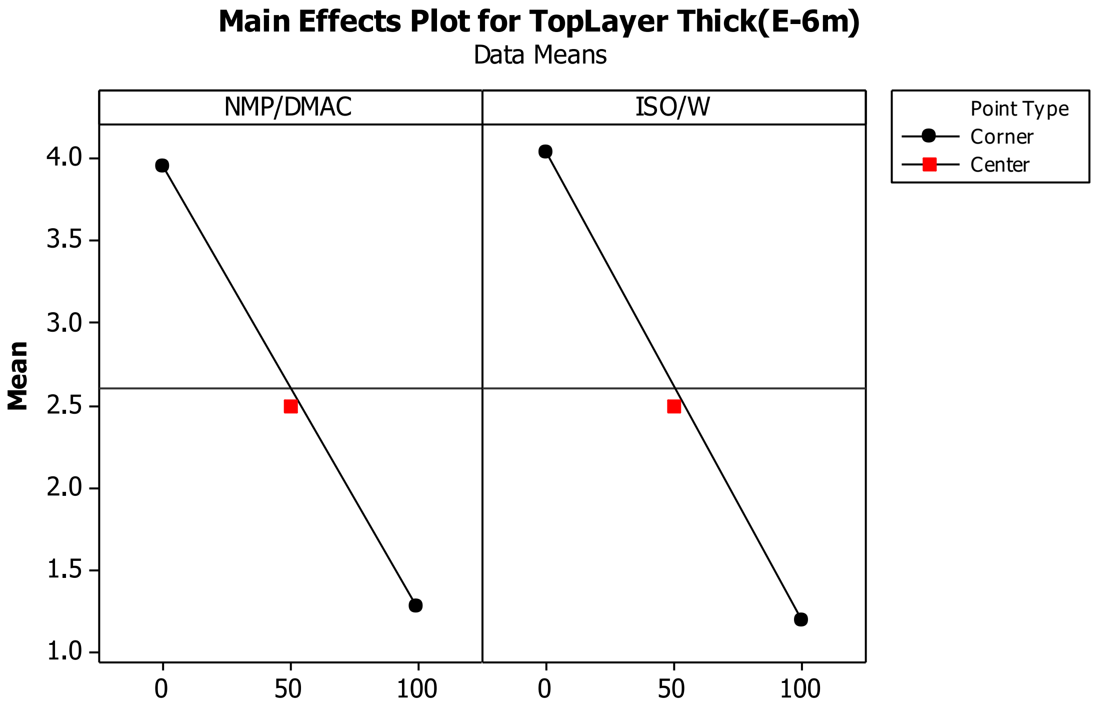

Figure A.8 Main effects plots of response (Top layer thickness)

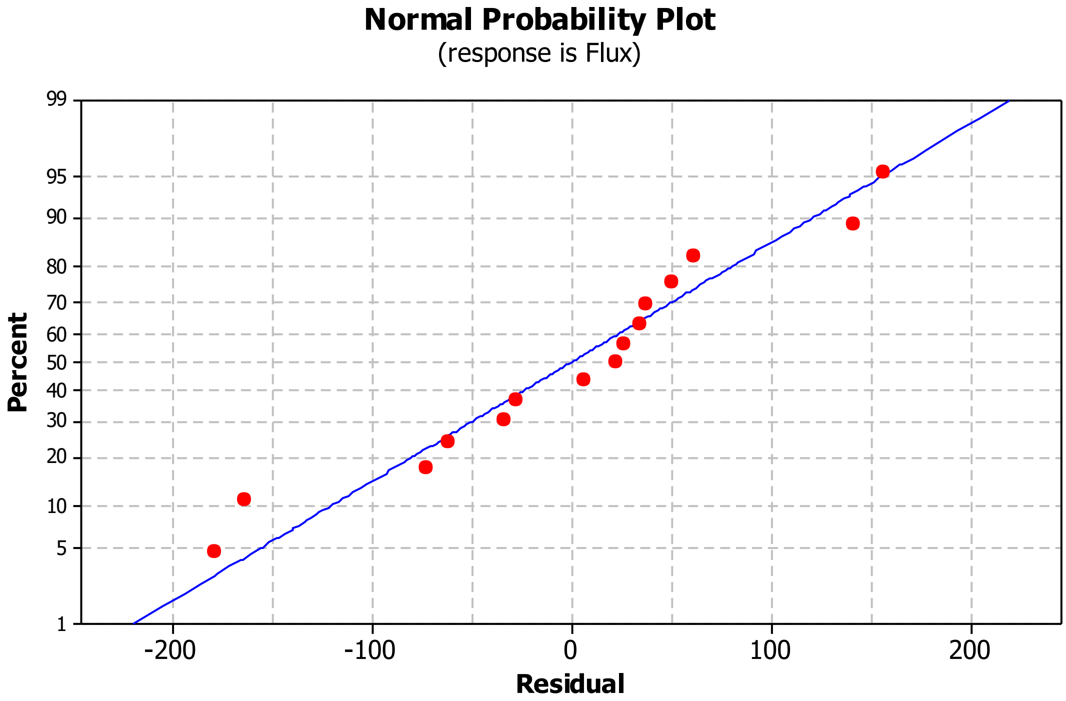

Figure A.9 Normal probability plot of residuals

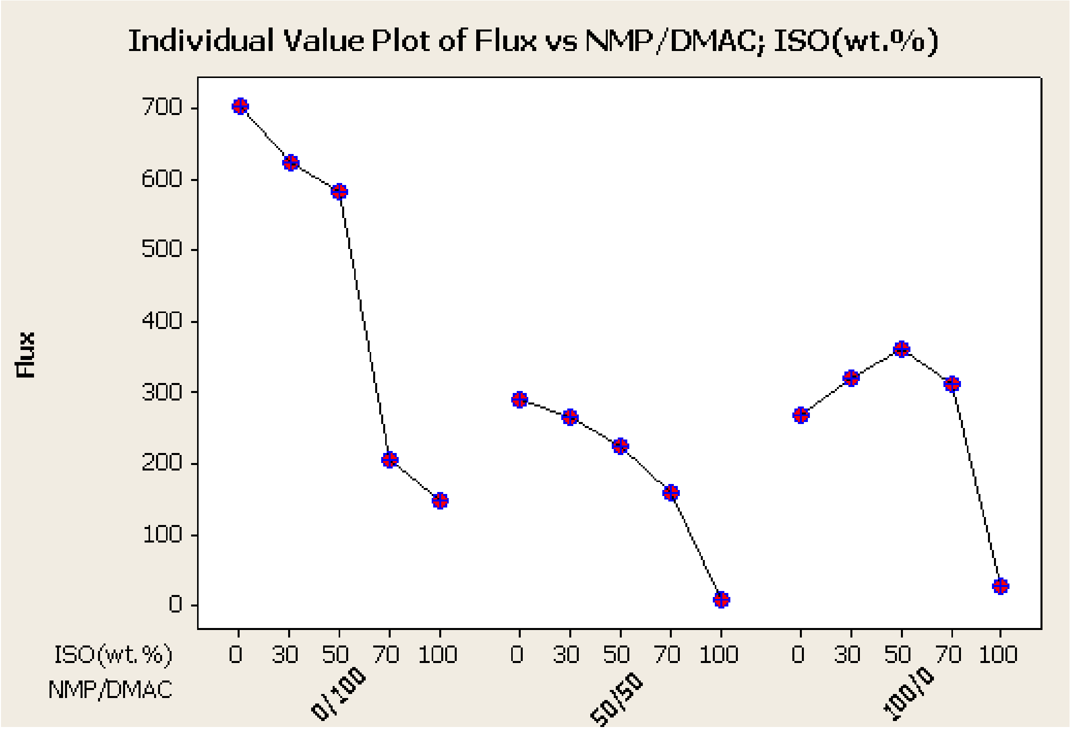

Figure A.10 Individual value plot of Flux (LMH) versus factors A and E

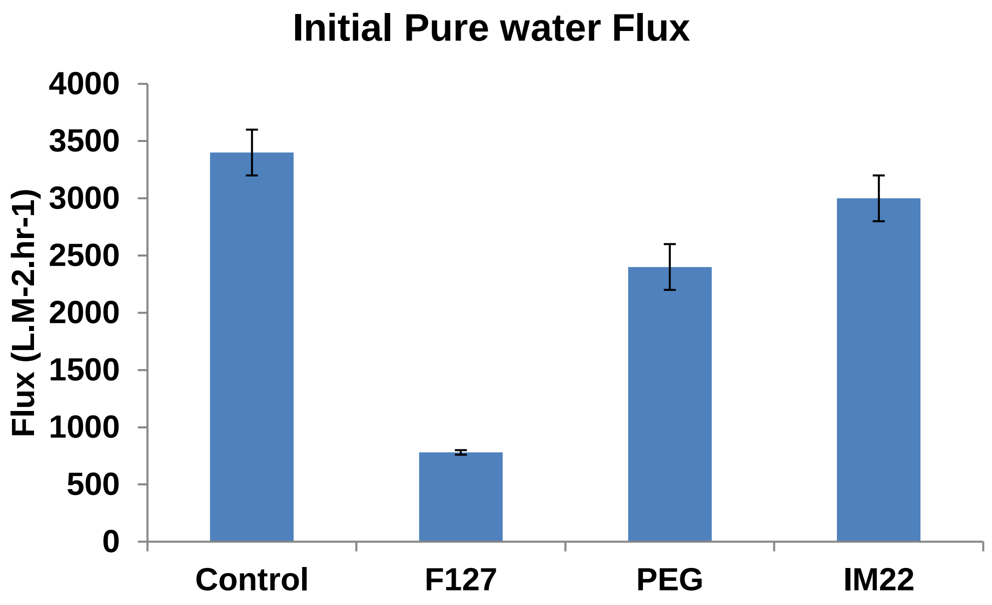

(a)

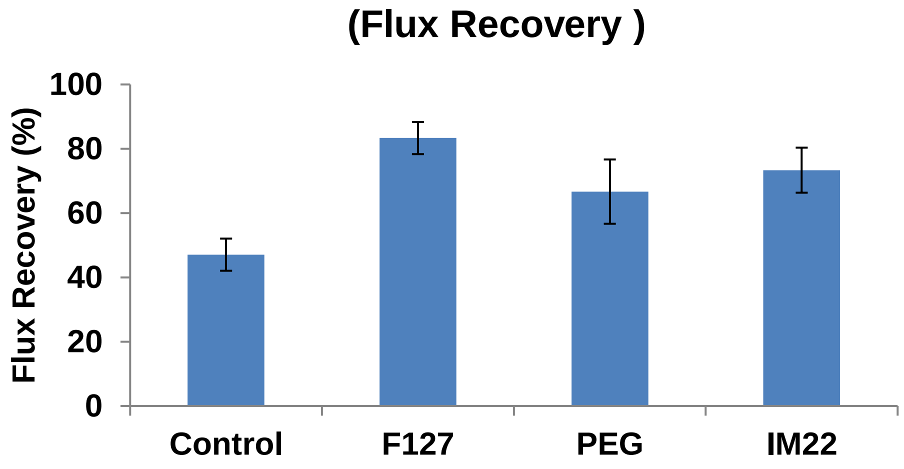

(b)

Figure A.11 (a) shows the pure water flux and flux recovery (b) of the membranes. As can be seen in the figure, the best surfactant to minimize the membrane fouling was F127.
